# Particle Acceleration Due to Coronal Non-null Magnetic Reconnection

**DOI:** 10.1007/s11207-017-1060-0

**Published:** 2017-03-14

**Authors:** James Threlfall, Thomas Neukirch, Clare Elizabeth Parnell

**Affiliations:** School of Mathematics and Statistics, Mathematical Institute, University of St Andrews, St Andrews, KY169SS UK

**Keywords:** Energetic particles, acceleration, Magnetic reconnection, observational signatures, Magnetic reconnection, theory, Magnetic fields, corona, Flares, relation to magnetic field

## Abstract

Various topological features, for example magnetic null points and separators, have been inferred as likely sites of magnetic reconnection and particle acceleration in the solar atmosphere. In fact, magnetic reconnection is not constrained to solely take place at or near such topological features and may also take place in the absence of such features. Studies of particle acceleration using non-topological reconnection experiments embedded in the solar atmosphere are uncommon. We aim to investigate and characterise particle behaviour in a model of magnetic reconnection which causes an arcade of solar coronal magnetic field to twist and form an erupting flux rope, crucially in the absence of any common topological features where reconnection is often thought to occur. We use a numerical scheme that evolves the gyro-averaged orbit equations of single electrons and protons in time and space, and simulate the gyromotion of particles in a fully analytical global field model. We observe and discuss how the magnetic and electric fields of the model and the initial conditions of each orbit may lead to acceleration of protons and electrons up to 2 MeV in energy (depending on model parameters). We describe the morphology of time-dependent acceleration and impact sites for each particle species and compare our findings to those recovered by topologically based studies of three-dimensional (3D) reconnection and particle acceleration. We also broadly compare aspects of our findings to general observational features typically seen during two-ribbon flare events.

## Introduction

Magnetic reconnection was first conceived as a way to explain the possible generation of high-energy particle populations during a solar flare (Giovanelli, 1946). Since then, it has been established as the fundamental way by which complex magnetic fields commonly restructure into a lower energy state. This restructuring also allows stored magnetic energy to be converted from initially complex field configurations into other forms of energy. Magnetic reconnection in three dimensions (3D) is also generically associated with parallel electric fields (indeed, the only necessary and sufficient condition for 3D reconnection to take place is that $\int E_{\|}\,ds\neq0$, see *e.g.* Schindler, Hesse, and Birn, 1988; Hesse and Schindler, 1988; Schindler, Hesse, and Birn, 1991, where $E_{\|}$ is the component of the electric field parallel to the magnetic field and $ds$ is a line element along which a component of the magnetic field persists). Direct acceleration through these electric fields at the primary energy release sites in the solar corona is likely to be a significant contributor to accelerated particle populations (as discussed in detail in *e.g.* Birn and Priest, 2007; Zharkova *et al.*, 2011).

A common approach in the study of magnetic reconnection is to use test particles. This is one of several methods that allow us to bridge the gap between the macroscopic description of a system undergoing magnetic reconnection and the kinetic scales of the plasma response. Other methods, for example particle-in-cell (or PIC), are better at describing the interplay between macroscopic and kinetic scales (*e.g.* Baumann, Haugbølle, and Nordlund, 2013) but due to numerical constraints, they can only be considered over a severely restricted size of the domain and also require rescaling of certain parameter regimes. Test particles, on the other hand, omit any back-reaction upon the global field (and each other) that is caused by their motion.

The equations that govern test-particle motion (for example guiding centre theory, *e.g.* Vandervoort, 1960; Northrop, 1963) are relatively well understood. Advances in computational power have allowed test-particle studies to move from simple idealised 2D configurations to fully 3D models of complex macroscopic configurations with reconnection taking place in different guises and at multiple sites. Many commonly focus on isolated topological features that are known to be likely sites of current sheet formation and reconnection in 3D, typically motivated by flare particle acceleration. Examples include several regimes of 3D null-point reconnection (*e.g.* Dalla and Browning, 2005, 2006, 2008; Guo *et al.*, 2010; Stanier, Browning, and Dalla, 2012), magnetic separator reconnection (*e.g.* Threlfall *et al.*, 2015, 2016a) or reconnection at fragmented current sheets (*e.g.* Turkmani *et al.*, 2005, 2006; Onofri, Isliker, and Vlahos, 2006; Gordovskyy, Browning, and Vekstein, 2010). The application of test-particle analysis to simulations of larger structures embedded in the solar atmosphere, including coronal loops (*e.g.* Gordovskyy *et al.*, 2014) or indeed entire active regions (Threlfall *et al.*, 2016b) has also uncovered evidence of significant particle acceleration. It is noteworthy that such large-scale structures often contain many locations and topological features (such as nulls, separatrix surfaces, spine lines, and separators) where reconnection takes place. Any resulting acceleration is also intrinsically linked to the chosen parameter regime, specifically depending on resolution and magnetic Reynolds number. When studying separator reconnection, Threlfall *et al.* (2016a) showed that simple analytical models reproduce all of the essential features of much more complex numerical separator reconnection models at a fraction of the computational effort. In addition, orbit calculation results based on such models can be rescaled without recalculation, allowing access to wider ranges of parameter space than models with specific numerical constraints.

In this present investigation, we examine test-particle behaviour in the vicinity of a simple analytical (scale-free) model of 3D magnetic reconnection without topological features (*e.g.* magnetic nulls or separators) associated with reconnection. The magnetic field in this model (based on Hesse, Forbes, and Birn, 2005) has similarities to that of an erupting magnetic flux rope. The primary objective of the present work is to investigate the overall response of test particles to this reconnection scenario, which is free of such topological features. Key questions that we seek to answer are how the particle acceleration in this model relates to other (topologically underpinned) models of magnetic reconnection and particle acceleration. Despite the simplicity of this model, we wish to determine whether the resulting particle orbits tie in with observational features that are typically associated with particle acceleration in the solar atmosphere, for example during a flare.

The article is organised as follows. In Section 2 we discuss the model itself, which combines a test-particle approach (whose governing equations are outlined in Section 2.1) with a simple kinematic global field that models the eruption of a magnetic flux tube due to (non-null) magnetic reconnection (described in Section 2.2). Our results are presented in Section 3, with analysis in Section 4 (including a comparison with other topologically-based models of magnetic reconnection in Section 4.1, and a broad comparison of our results with aspects of solar flare observations in Section 4.2). We finally outline our conclusions and future areas of study in Section 5.

## Model Setup

Our approach is broadly split into two components. The guiding centre test-particle orbit motion equations, which are solved numerically for a given set of electric and magnetic fields, form the first component. The second component is an analytical global model that describes a reconnection event in the absence of magnetic null points (in the context of the eruption of a magnetic flux rope) used by the orbit calculations. A brief overview of each component follows.

### Relativistic Particle Dynamics

We first outline the equations that govern the particle behaviour. Our investigation makes use of the full relativistic set of guiding-centre-motion equations, outlined in Northrop (1963), which we briefly recapitulate here:

1 $$\begin{aligned} \frac{d{u_{\parallel}}}{dt} =&\frac{d}{dt} (\gamma v_{\parallel} )=\gamma {{ \mathbf{u}}_{E}}\cdot{\frac{d{\mathbf{b}}}{dt}}+{{\Omega _{\mathrm{scl}}}} {t_{\mathrm{scl}}}E_{\parallel}-\frac{\mu _{r}}{\gamma}\frac{\partial{B^{\star}}}{\partial s}, \end{aligned}$$

2 $$\begin{aligned} \dot{\mathbf{R}}_{\perp} =&{{\mathbf{u}}_{E}}+\frac{\mathbf{b}}{B^{\star\star }}\times \biggl\lbrace \frac{{1}}{{{\Omega_{\mathrm{scl}}}}{t_{\mathrm{scl}}}} \biggl[ \frac{\mu _{r}}{\gamma} \biggl(\boldsymbol{\nabla} {B^{\star}}+ \frac{{{v_{\mathrm{scl}}}^{2}}}{{c^{2}}}{{\mathbf{u}}_{E}} \frac{\partial B^{\star}}{\partial t} \biggr) \\ &{} +u_{\parallel}\frac {d{\mathbf {b}}}{dt}+\gamma\frac{d{{\mathbf{u}}_{E}}}{dt} \biggr]+\frac {{{v_{\mathrm{scl}}}^{2}}}{{c^{2}}} \frac {u_{\parallel}}{\gamma}{E_{\parallel}} {{\mathbf{u}}_{E}} \biggr\rbrace , \end{aligned}$$

3 $$\begin{aligned} \frac{d\gamma}{dt} =&\frac{{{v_{\mathrm{scl}}}^{2}}}{{c^{2}}} \biggl[{{\Omega _{\mathrm{scl}}}} {t_{\mathrm{scl}}} \biggl(\dot{\mathbf{R}}_{\perp}+\frac{u_{\parallel}}{\gamma}{\mathbf{b}} \biggr)\cdot {\mathbf {E}}+\frac{\mu_{r}}{\gamma}\frac{\partial B^{\star}}{\partial t} \biggr], \end{aligned}$$

4 $$\begin{aligned} \mu_{r} =&\frac{\gamma^{2}{v_{\perp} ^{2}}}{B}. \end{aligned}$$

We note that $\mu_{r}$ is the relativistic magnetic moment for a particle with rest mass $m_{0}$ and charge $q$, whose guiding centre is located at ${\mathbf{R}}$, subject to an electric field ${\mathbf{E}}$ and a magnetic field ${\mathbf{B}}$ with magnitude $B=|{\mathbf{B}}|$ and unit vector $\mathbf{b}={\mathbf{B}}/B$. Local conditions will dictate aspects of the orbit behaviour, particularly through guiding-centre drifts; the largest in magnitude is typically the ${E}\times{B}$ drift, which has a velocity ${\mathbf{u}}_{E}=\mathbf{E}^{\star}\times{\mathbf{b}}/B$. The component of velocity parallel to the magnetic field is $v_{\parallel} =\mathbf {b}\cdot{\dot{\mathbf{R}}}$, while $E_{\parallel}=\mathbf{b}\cdot{\mathbf{E}}$ is the magnitude of the electric field parallel to the local magnetic field, $\dot{\mathbf{R}}_{\perp}= \dot{\mathbf{R}}-v_{\parallel} {\mathbf{b}}$ is the component of the velocity perpendicular to ${\mathbf{b}}$, and $s$ is a line element parallel to ${\mathbf{b}}$. Finally, $\gamma$ is the Lorentz factor, relating velocities to the speed of light through $\gamma^{2}=1/ (1-v^{2}/c^{2} )$. Using this factor, we define a relativistic parallel velocity $u_{\parallel}=\gamma v_{\parallel} $ for simplicity of notation.

For a given magnetic field strength $B$, $B^{\star}$ and $B^{\star \star }$ are defined as

$$B^{\star}=B \biggl( 1-\frac{1}{c^{2}}\frac{{E_{\perp} }^{2}}{B^{2}} \biggr)^{\frac {1}{2}} , \qquad B^{\star\star}=B \biggl(1-\frac{1}{c^{2}} \frac {{E_{\perp} }^{2}}{B^{2}} \biggr). $$

The multiplying quantities are dimensionless, *i.e.* $B^{\star}$ and $B^{\star\star}$ retain the dimensions of the magnetic field.

Equations (1) – (4) are dimensionless, and related to physical quantities by multiplying the relevant non-dimensional quantity by a magnetic field strength ${b_{\mathrm{scl}}}$, a length-scale ${l_{\mathrm{scl}}}$, a timescale ${t_{\mathrm{scl}}}$, or some combination thereof, *e.g.*:

$${\mathbf{B}}={b_{\mathrm{scl}}}\,\bar{\mathbf{B}}, \qquad x={l_{\mathrm{scl}}}\,\bar{x}, \qquad t={t_{\mathrm{scl}}}\, \bar{t}, $$

where the barred quantities represent dimensionless counterparts of specific variables. The choice of these quantities fixes the remaining normalising constants. Our normalising parameters are chosen so that the resulting particle behaviour may reflect the behaviour found in the solar corona; hence, unless otherwise stated, all experiments take ${b_{\mathrm{scl}}}=0.001~\mbox{T}$, ${l_{\mathrm{scl}}}=100~\mbox{km}$ and ${t_{\mathrm{scl}}}=100~\mbox{s}$.

The guiding-centre equations (Equations (1) – (4)) are further simplified by considering only electrons or protons in this study. The rest mass $m_{0}=m_{e}=9.1\times10^{-31}~\mbox{kg}$ and charge $q=e=-1.6022\times10^{-19}~\mbox{C}$ are fixed for electrons, or $m_{0}=m_{p}=1.67\times10^{-27}~\mbox{kg}$ and $q=|e|=1.6022\times 10^{-19}~\mbox{C}$ for protons. This allows us to express several normalising constants in terms of a normalising electron or proton gyrofrequency, $\Omega_{\mathrm{scl}}={q\,{b_{\mathrm{scl}}}}{m_{0}}^{-1}$, which controls the scales at which certain guiding-centre drifts become important.

The guiding-centre equations (1) – (4) are evolved in time using a fourth-order Runga–Kutta scheme, subject to the global electric and magnetic fields in our model. Errors are minimised through the use of a variable time step, constrained by comparisons of the fourth- and fifth-order Runga–Kutta calculations at each step. This scheme has been directly used in several recent works (*e.g.* Threlfall *et al.*, 2015, 2016a,b; Borissov, Neukirch, and Threlfall, 2016), with similar implementations in other recent investigations (*e.g.* Gordovskyy *et al.*, 2014). By controlling the timescales of the guiding-centre approximation code and the timescale of the global flux-rope eruption model so that they never become of a similar size, we are justified in our use of this test-particle approach. Similarly, we monitor the spatial scales of the gyromotion and the global environment, in order to ensure that they remain disparate as well. However, in order to proceed, we must now define the environment in which the particles will gyrate.

### Non-null Magnetic Reconnection Model

For this study, we modify a simple analytical kinematic model first proposed by Hesse, Forbes, and Birn (2005) which aims to describe how an arcade of closed sheared magnetic field lines may reconnect to form helical field lines and mimic the evolution and eruption of a flux rope within the solar atmosphere.

To begin, we create a magnetic vector potential, ${\mathbf{A}}$, of the form

5 $$ {\mathbf{A}} (t )=b_{0}l_{0} \biggl[- \biggl(1+ \frac{\epsilon (t)\bar {z}}{ (1+\frac{\bar{z}^{2}}{{\bar{L_{z}}}^{2}} ) (1+\frac {\bar {x}^{2}}{{\bar{L_{x}}}^{2}} )} \biggr){\hat{\mathbf{x}}} + \frac{\bar {x}\bar{y}}{\bar {L_{y}}}{\hat{\mathbf{y}}} + ( \bar{x}+0.2\bar{y} ){\hat{\mathbf{z}}} \biggr], $$

for a magnetic field strength $b_{0}$ and length scale $l_{0}$ using Cartesian variables expressed in non-dimensional form. From this point on, we endeavour to use real (rather than normalised) variables and set $l_{0}={l_{\mathrm{scl}}}$ and $b_{0}={b_{\mathrm{scl}}}$ throughout. Equation (5) fully defines the electric field in the model (in the absence of an electrostatic potential). We note that a magnetic vector potential was not specified in the original model of Hesse, Forbes, and Birn (2005). The constant in the $z$-component (here set to 0.2) is arbitrary. This value defines the level of shear in the magnetic field resulting from $\boldsymbol{\nabla} \times{\mathbf{A}}$. We have also rotated the original coordinate system of the magnetic field specified in Hesse, Forbes, and Birn (2005). In our model, the $z$-axis is aligned with the vertical direction (perpendicular to the solar surface). The model photosphere lies in the $xy$-plane where $z=0$. The model includes a spatial perturbation, controlled by a time-dependent parameter, $\epsilon(t)$. When combined, these factors control how the flux rope and electric fields evolve. We select a form for the time-dependence once we have considered how the spatial and temporal parts of the perturbation appear in the equations for the electric and magnetic field.

The resulting magnetic field is

6 $$\begin{aligned} {\mathbf{B}}=\boldsymbol{\nabla} \times{\mathbf{A}}=b_{0} \biggl[0.2{\hat{\mathbf{x}}} - \biggl(1+\frac{\epsilon (t) (1-\frac{{z}^{2}}{{L_{z}}^{2}} )}{ (1+\frac {{z}^{2}}{{L_{z}}^{2}} )^{2} (1+\frac{{x}^{2}}{{L_{x}}^{2}} )} \biggr){\hat{\mathbf{y}}} + \frac{{y}}{L_{y}}{\hat{\mathbf{z}}} \biggr]. \end{aligned}$$

Parameters $L_{x}$, $L_{y}$, and $L_{z}$ anchor the model at a specific location and have the same dimensions as $x$, $y$, and $z$ (hence, $L_{x}=l_{0}\bar{L_{x}}$, *etc.*). Equation (6) describes a series of sheared arcade-like field lines at $\epsilon(t)=0$, which then reconnect due to the addition of a (circuital) magnetic field perturbation. The form of this perturbation is chosen to reconnect both short magnetic loops and the overlying helical field; in this way, this simple model reproduces the basic effects that are thought to occur during the eruption of a flux rope. As noted in Hesse, Forbes, and Birn (2005), this field is non-vanishing everywhere and does not contain topological features, which are often considered as likely sites of magnetic reconnection, including magnetic null-points, separators, or separatrix surfaces.

The addition of the time-varying perturbation $\epsilon$ also generates an electric field through Faraday’s law, in the absence of an electrostatic potential, *i.e.*

7 $$ {\mathbf{E}}=-\frac{d{\mathbf{A}}}{dt}=\frac{b_{0}z}{{ (1+\frac {z^{2}}{{L_{z}}^{2}} ) (1+\frac{x^{2}}{{L_{x}}^{2}} )}}\frac {d{\epsilon}}{dt}{\hat{\mathbf{x}}}. $$

**Figure 1:**
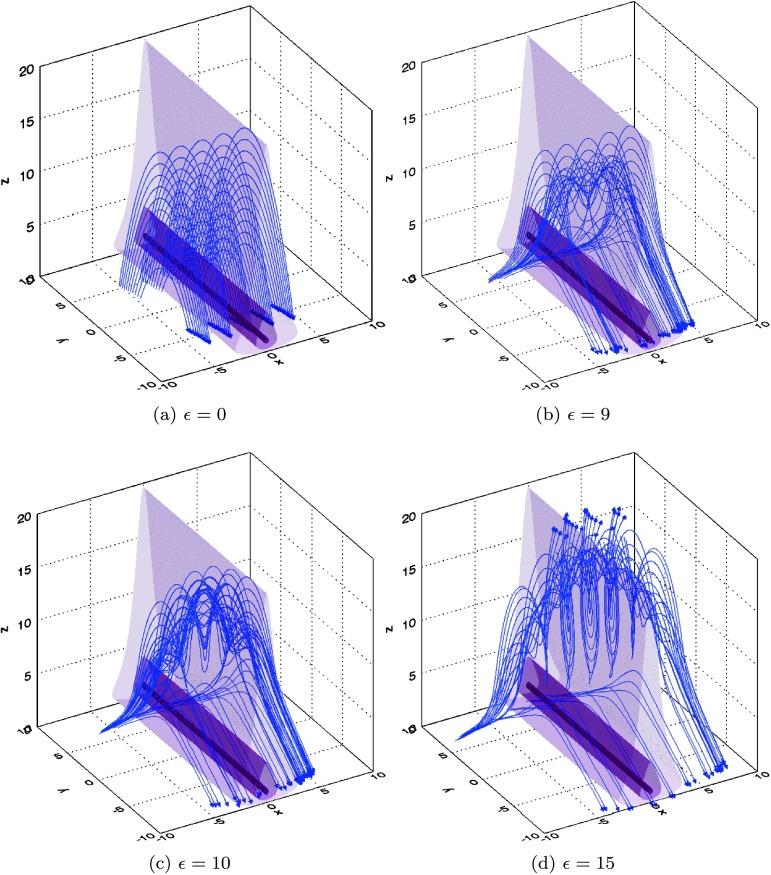
Evolution of (non-dimensional) magnetic fields and isosurfaces of the electric field strength in our chosen model, outlined in Equations (5) – (7), seen at different stages of the flux rope eruption. The magnetic field is illustrated by blue field lines (with arrows denoting orientation), while purple contours show the strength of the electric field (opacity at 10, 25, and $50~\%$ representing the same percentage of $E_{\mathrm{max}}$, which reaches $50~\mbox{V}\,\mbox{m}^{-1}$ when ${l_{\mathrm{scl}}}=100~\mbox{km}$, ${b_{\mathrm{scl}}} =0.001~\mbox{T}$, and ${t_{\mathrm{scl}}}=100~\mbox{s}$, for example). In the case shown here, $\bar{L_{x}}=5\sqrt{2}$, $\bar{L_{y}}=\bar{L_{z}}=5$ in a region $\bar{x},\bar{y}\in[-10,10]$, $\bar{z}\in[0,20]$.

The strength of the electric field depends on the chosen form of $\epsilon(t)$; with our photosphere at $z=0$, the electric field increases linearly with height above the photosphere before decreasing much more slowly above a height determined by $L_{z}$. We illustrate the behaviour of both the magnetic field and the strength of the electric field outlined here at different values of $\epsilon$ (which may also be thought of as being at different times) in Figure 1.

By considering $\epsilon=t/\tau$ for a constant global timescale, $\tau $, we recover an electric field that is constant in time. We also note the findings of Hesse, Forbes, and Birn (2005) regarding this model, in which the flux rope only begins to form at $\epsilon\approx10$. By setting $\tau={t_{\mathrm{scl}}}$ (our normalisation time for the particle orbit code) and initialising particles at integer multiples of ${t_{\mathrm{scl}}}$ (so that $t=0,{t_{\mathrm{scl}}}, 2{t_{\mathrm{scl}}}$, *etc.*), we automatically evolve the global model on much longer timescales than those over which the particles gyrate (recalling that ${t_{\mathrm{scl}}}=100~\mbox{s}$, while a typical gyroperiod is of the order of milliseconds or shorter).

We want to emphasise again that the key aspect of this model is that it is fully analytical and that the details of the reconnection can be tuned to match desired properties, *e.g.* to match observations or simulations. In this investigation, we hope to gain a general sense of a particle response to a simple flux rope eruption event, using a configuration that does not contain nulls or other topological features of interest. This would then provide a benchmark for later, more complex environments.

## Orbit Calculation Results

In order to study different aspects of the particle response to this model, we analysed more than 8000 particle orbit calculations based on two different sets of test-particle initial conditions. We outline these in Section 3.1, before describing the resulting final orbit positions (Section 3.2), energies, and spectra (Section 3.3) recovered using the two sets of initial conditions. The conditions of the global model remain fixed regardless of the choice of particle initial conditions. We choose global parameters $L_{x}=5\sqrt{2}{l_{\mathrm{scl}}}\approx707~\mbox{km}$ and $L_{y}=L_{z}=5{l_{\mathrm{scl}}} =500~\mbox{km}$ (corresponding to a peak electric field of $50~\mbox{V}\,\mbox{m}^{-1}$ at $z=500~\mbox{km}$, $x=0~\mbox{km}$) for ${l_{\mathrm{scl}}}=100~\mbox{km}$. We only studied the region in the vicinity of the flux rope expansion and eruption, using the range $xy\in[-10,10]{l_{\mathrm{scl}}}$ and $z\in [0,40]{l_{\mathrm{scl}}}$ (*i.e.* $xy\in[-1,1]~\mbox{Mm}$ and $z\in [0,4]~\mbox{Mm}$ when ${l_{\mathrm{scl}}}=100~\mbox{km}$) for all test-particle calculations. We analysed the behaviour of a new set of particles at different times over the course of the evolution of the global magnetic field. With ${t_{\mathrm{scl}}}=100~\mbox{s}$, and $\epsilon=t/\tau=t/{t_{\mathrm{scl}}}$, we initialised a new set of particles every $100~\mbox{s}$; this corresponds to initialisation of orbits at integer values of $\epsilon$.

### Test-Particle Initial Conditions

**Figure 2:**
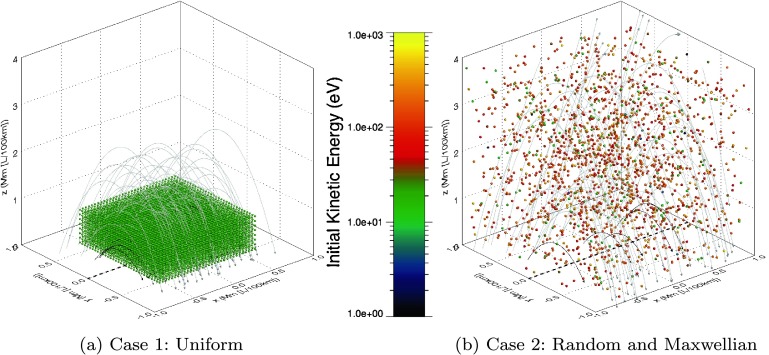
Initial conditions used in our experiments. (a) Corresponds to Case 1, which uses a uniform grid of initial particle positions all at $20~\mbox{eV}$ kinetic energy and $45^{\circ}$ pitch angles. (b) Shows a Case 2 example, where initial positions and pitch angles are randomised, with particle energies selected according to a Maxwellian distribution. Each orb in either figure represents an initial particle position and is colour coded according to the initial particle energy (see colour bar). The field configuration at $\epsilon=0$ has been included for reference, with the overlying field identified by grey lines and the low-lying field by black field lines.

To study different aspects of the model behaviour, we used two different sets of initial conditions for the orbits, which we label Case 1 or Case 2. A visual representation of these cases (and the initial field structure of the global model) is provided in Figure 2.

Case 1 comprises an evenly spaced grid of initial orbit positions containing particles with identical initial kinetic energy and pitch angle. Such a uniformly spaced initial set of positions is physically unrealistic, but allows us to build up a clear picture of the particle response to the reconnection region and its surroundings. A total of 8192 particles are divided evenly between eight separate $z$-planes (at 0.1, 0.2, 0.3, 0.4, 0.5, 0.6, 0.7, and 0.8 Mm above the photosphere) and are distributed in a $32\times32$ grid in ($x$, $y$) (ranging from $-0.6\rightarrow0.6~\mbox{Mm}$). Each orbit begins with an initial pitch angle of $45^{\circ}$ and $20~\mbox{eV}$ of kinetic energy. These values are deliberately chosen to limit the initial parallel velocity of each orbit and establish general particle behaviour trends within the reconnection region. Case 1 initial conditions are illustrated by Figure 2a.

Owing to the importance of the energies that result from our calculations, Case 2 comprises a more “realistic” set of initial conditions. In Case 2, the initial orbit energies adhere to a Maxwellian distribution that peaks at an approximately coronal temperature (1 MK). These orbits are also given random initial positions throughout the domain and random initial pitch angles. An example of the initial conditions found in Case 2 is illustrated in Figure 2b. We also note that earlier investigations (*e.g.* Threlfall *et al.*, 2015) showed that local parallel electric fields (if present) completely dominate the orbit energetics of test-particle orbits, but these investigations have so far only used simplistic (uniform) initial conditions. One of our objectives here is to show how our results are affected by the choice of initial conditions.

### Final Orbit Positions

**Figure 3:**
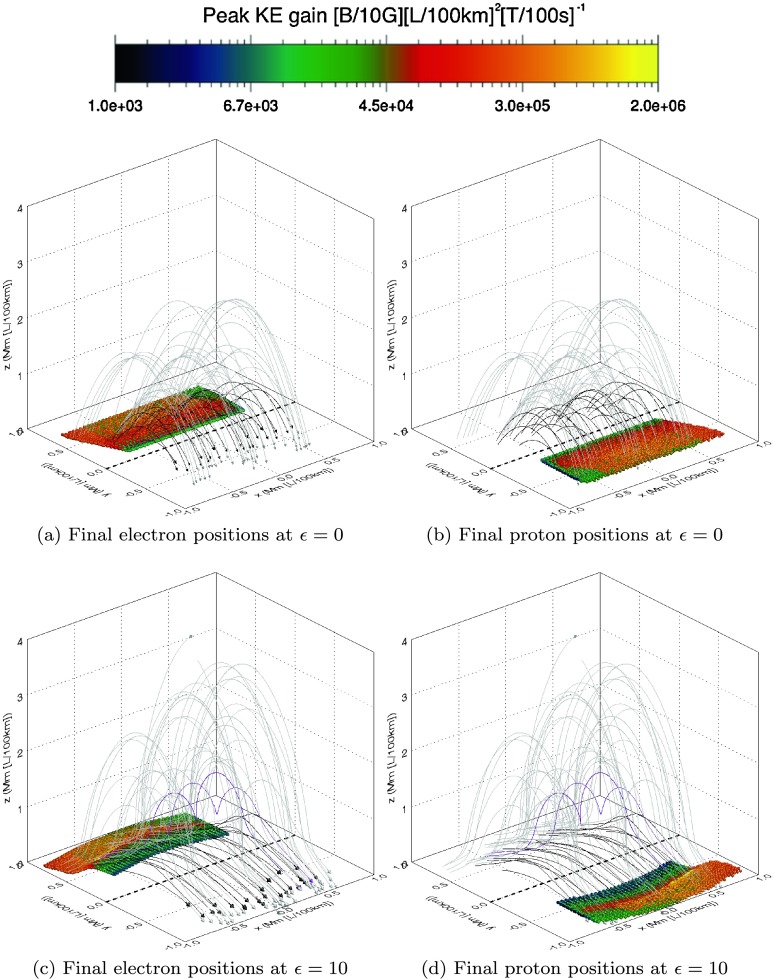

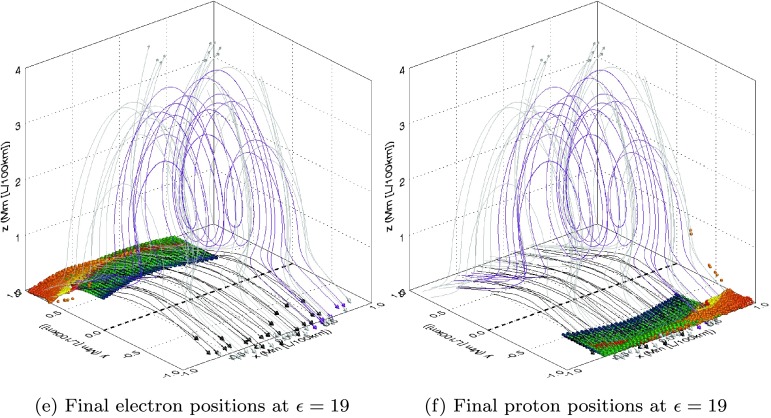
(a), (c), and (e) Correspond to the final electron, while (b), (d), and (f) show the final proton positions, colour coded according to the peak kinetic energy gain (see legend). Each image is overlaid with selected magnetic field lines illustrating the current stage of the magnetic flux rope eruption: black lines indicate low-lying field, purple indicates helical field lines, and grey indicates overlying field. The dashed black line highlights the location of the polarity inversion line (PIL).

The final positions of each of the orbits recovered using Case 1 and Case 2 are very similar, but those for Case 2 are somewhat complicated by the random initial positions. For simplicity, we describe the key findings for this aspect of our study using the initial conditions of Case 1. Figure 3 illustrates the final positions of electron and proton orbits using the initial conditions of Case 1 at different values of $\epsilon$ (which approximately correspond to the start, middle, and end of the flux rope formation in our model). The left column of this figure illustrates the electron response, while the right column illustrates the proton response. Each final position is colour coded according to the peak energy gained over the course of the orbit lifetime. In order to provide context to the final orbit positions, magnetic field lines are plotted in Figures 3a – 3b of the global magnetic structure at $\epsilon=0$, while Figures 3c –  3d illustrate the stage when the twisted flux rope becomes visible (at $\epsilon=10$), Figures 3e – 3f show the developed flux rope at the end of the experiment ($\epsilon=19$). For clarity, we have chosen to colour field lines in the global field model depending on their trajectories. Black field lines denote a magnetic field that never rises above $z=0.75~\mbox{Mm}$. Purple lines indicate helical magnetic field lines (which, for the purposes of identification, we define to be lines that contain more than one maxima in $z$). Grey lines denote a magnetic field that is not helical and reaches above $0.75~\mbox{Mm}$ (and typically forms overlying parts of the magnetic arcade). Thus the appearance of increasing numbers of purple field lines in each row of Figure 3 implies that the global field passing through the reconnection region is becoming more helical as time progresses.

All of the orbits in Figure 3 terminate upon impact with the edge of our artificial domain. This domain is arbitrary, but encompasses and extends beyond the reconnection region. No orbits show evidence that they are mirrored by the magnetic mirror effect (where an orbit entering a region of increasing magnetic field strength may be reflected by the invariance of the magnetic moment).

Before the flux rope is formed, the first row of Figure 3 shows that all electrons are accelerated along the sheared magnetic arcade towards the positive $y$-region of the photosphere, while protons are accelerated towards the negative $y$-region of the photosphere (due to the difference in charge). Lower energy electrons typically impact the photosphere closer to the polarity inversion line (PIL), shown at the base of each image as a dashed black line along $y=0$. These electrons travel along short field lines that do not extend high into the solar atmosphere. Higher energy electrons reach higher into the atmosphere and terminate farther from the PIL in $y$ while they are being spread almost uniformly in the $x$-direction (with a small minority terminating predominantly in the negative $x$-quadrant). The proton results (Figure 3b) display similar trends, but with all orbits accelerated in the *opposite* direction (towards the negative $y$-quadrant of the photosphere) and with the same minor asymmetry in $x$ (where highly accelerated protons appear only slightly more likely to impact the solar surface in the positive $x$-quadrant). This minor asymmetry is most likely the result of the original shear imposed upon the magnetic field.

As time progresses and the flux rope starts to develop, this minor asymmetry in the spread of protons and electrons parallel to the PIL develops further. At $\epsilon=10$, the final electron positions seen in the second row of Figure 3 are farther from the PIL in $y$, with many more strongly accelerated electrons now arriving in the negative $x$-quadrant than seen at $\epsilon=0$. Many more protons (Figure 3d) also terminate in the positive $x$-region, while the entire distribution of final positions again lies at an equal distance from the PIL in $y$ as the electrons, but on the opposite side.

The asymmetry in the final position parallel to the PIL is extremely pronounced by $\epsilon=19$. The final positions of electrons (Figure 3e) and protons (Figure 3f) fall into two highly distinct categories. Orbits with weak energy gains uniformly terminate at the photosphere in a region $|x|\leq0.6~\mbox{Mm}$ and with $y\geq0.4~\mbox{Mm}$ for electrons and $y\leq-0.4~\mbox{Mm}$ for protons. Strongly accelerated orbits are exclusively found in the $(x<0, y>0)$ quadrant for electrons and in the $(x>0,y<0)$ quadrant for protons. At this stage of the experiment, many orbits impact with the side walls of our domain (which was not the case for earlier stages seen in the other rows of Figure 3).

**Figure 4:**
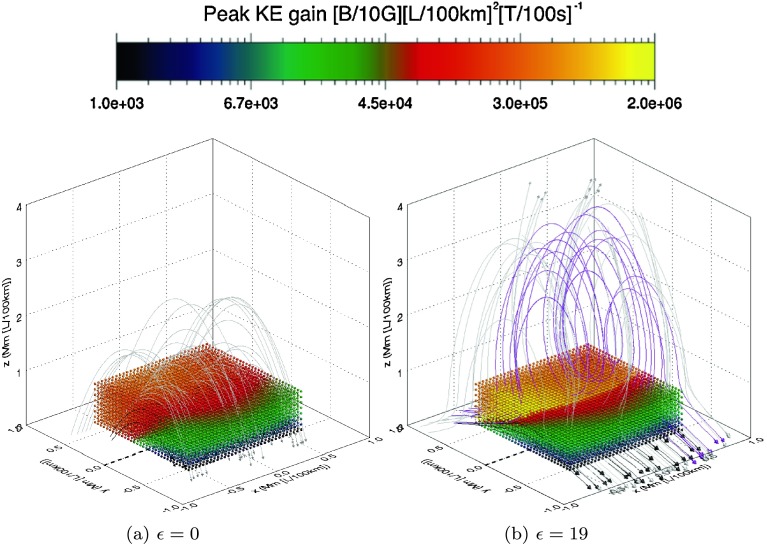
Initial proton positions colour coded according to peak kinetic energy gain (see legend) at (a) $\epsilon =0$ and (b) $\epsilon=19$. Also included are low-lying field lines (black), helical field lines (purple), and overlying field (grey). The dashed black line represents the PIL.

We are also able to relate regions of strongly or weakly accelerated orbits with specific initial locations using Figure 4, which maps the peak kinetic energy gain over the orbit lifetime back onto the initial particle position. For brevity, we only discuss the proton results at the initial and final stages of the global eruption model (as the electron results typically mirror the proton results in all cases and only differ in direction of acceleration and time taken to impact the domain boundary). Firstly, note that all particles at initial positions $(x_{0},y,z_{0})$ experience the same initial (parallel) electric field regardless of their $y$ coordinate. Thus all these particles start to accelerate in the same direction. This acceleration causes those protons (electrons) starting with $y>0$ ($y<0$) to travel along the field lines through an extended region on a non-zero parallel electric field, allowing them to be accelerated further and to gain higher energies. On the other hand, the protons (electrons) starting with $y<0$ ($y>0$) just travel away from the acceleration region and therefore only achieve low energies.

Additionally, we observe that the largest energy gains are strongly correlated to the locations where highly twisted helical field lines form. At $\epsilon=0$, no yellow positions exist in Figure 4a, indicating that the configuration is incapable of producing the largest energy gains at this time. These energies are only recovered at the later stages of the experiment. Since the electric field in our experiment is independent of time, the particles that achieve these higher energies must have done so by spending more time in the acceleration zone. Specifically, yellow orbits only begin to appear when the first helical field lines form, *e.g.* at $\epsilon =10$. The initial positions of these high-energy orbits are well aligned to the purple field lines, but associate with opposite ends of the flux rope depending on the particle species. This can be seen when $\epsilon =19$ in Figure 4b. The size of the region that produces the most highly accelerated orbits increases with height and also with time. By the time $\epsilon=19$, more reconnected helical field allows additional particles to enter the region where $E_{\|}$ is strongest and remain for longer, leading to acceleration to higher energies.

### Orbit Energies and Spectra

After broadly describing the impact and acceleration sites of orbits in this reconnection model, we now describe the acceleration and energy gains.

In this section, we would first clarify that we report specific orbit findings that are not based on particle fluxes and that should not be linked to observational spectra without great care. These spectra simply reflect the response of our specific particle population to the global environment according to their chosen initial conditions. The energetics are intrinsically linked to our choice of initial conditions and are limited by the fact that we are sampling a discrete set of orbits over a limited range of parameter space for initial position, energy, and pitch angle in both Cases 1 and 2. To illustrate this point, we note for example that the region of smallest energy gains in the bottom plane of Figure 4 actually grows with time. Average energies in this plane decrease from $102~\mbox{keV}$ at $\epsilon=0$ to $35.5~\mbox{keV}$ ($\epsilon=10$) and ultimately to $7.79~\mbox{keV}$ ($\epsilon=19$). This finding is misleading, however, as sampling the entire population reveals an overall increase in average kinetic energy with time. Thus orbits that sample a different range of initial parameter space may have significant effects on the energies and resulting spectra.

While the lowest plane of initial positions suffers a reduction in energy gained in Case 1, as the experiment progresses, the uppermost $z$-plane is able to achieve much higher energy gains. At $\epsilon=0$, the average electron energy gain in the top plane is approximately $155~\mbox{keV}$ (orange orbits in Figure 4a). This average value increases to $289~\mbox{keV}$ and $369~\mbox{keV}$ at $\epsilon=10$ and $\epsilon=19$, respectively. For identical initial conditions, both electrons and protons typically recover the same final energy (to five significant figures or better).

**Figure 5:**
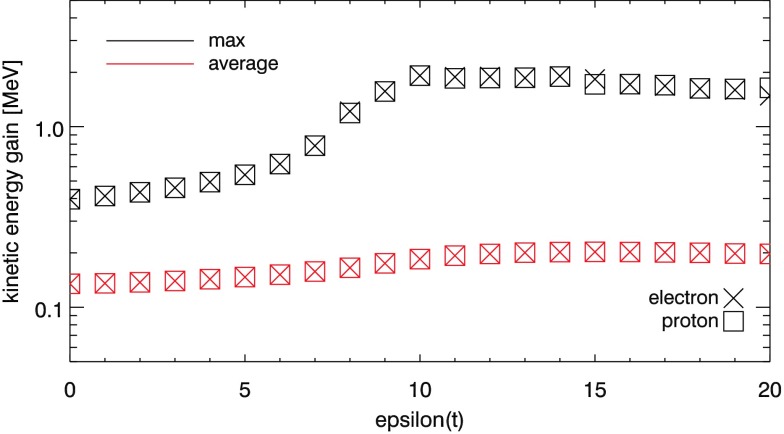
Peak and average electron and proton energy gains as a function of time ($\epsilon$) for particles with $20~\mbox{eV}$ initial kinetic energy and the initial grid outlined earlier. See insets for explanations.

In order to more fully describe the energetic impact of the “eruption” (formation and expansion of the flux rope) on the particle orbits, Figure 5 illustrates how the maximum and average kinetic energy gained in the experiment by all the particles for Case 1 changes with time. At $\epsilon=0$, the maximum kinetic energy gained by any orbit is $0.396~\mbox{MeV}$, while the average kinetic energy gained for all electrons or protons is $0.135~\mbox{MeV}$. Figure 5 allows us to broadly categorise the experiment as having three stages. The first stage begins at $\epsilon=0$ and sees a slow rise in both the average and peak kinetic energies of the orbits until $\epsilon\approx6$, where the maximum value has risen to $0.620~\mbox{MeV}$ and the average has risen to $0.151~\mbox{MeV}$. The second stage is marked by a much steeper, sustained increase in the maximum orbit energy gain, peaking at $1.92~\mbox{MeV}$ by $\epsilon=10$, accompanied by a smaller rise in average orbit energy (to $0.184~\mbox{MeV}$). Following $\epsilon\approx11$, the third stage sees a small decrease in peak energy gains, while the average energies remain at similar levels. At the end of the experiment, the maximum and average energy gains for Case 1 orbits are $1.59~\mbox{MeV}$ and $0.198~\mbox{MeV}$, respectively. Figure 5 also demonstrates that for the vast majority of the experiment, both protons and electrons yield nearly identical maximum and average kinetic energy gains at each stage of the global field evolution.

The three stages illustrated by this experiment again reflect the evolution of the global kinematic model. Following $\epsilon=0$, the footpoints of the sheared arcade widen. Strongly accelerated orbits are still accelerating as they reach the photosphere. Reaching the photosphere terminates the orbit calculation. Footpoint widening acts to slightly increase the amount of time each orbit has to gain energy through direct acceleration. The second observed stage of behaviour coincides with the first visual identification of the formation of the flux rope. Once formed, the flux rope allows additional field lines to repeatedly re-enter the reconnection region and again experience direct acceleration by the regions of strongest electric field. The third stage sees the peak and average energies remain almost constant, with perhaps a small decline, which could be due to the footpoint separation. In the final stages of the experiment, most orbits terminate at the side boundaries of our artificial domain, rather than at the photosphere. Orbits at later stages may therefore traverse slightly shorter field lines as $\epsilon$ increases, again raising the possibility of slightly limiting the acceleration process.

**Figure 6:**
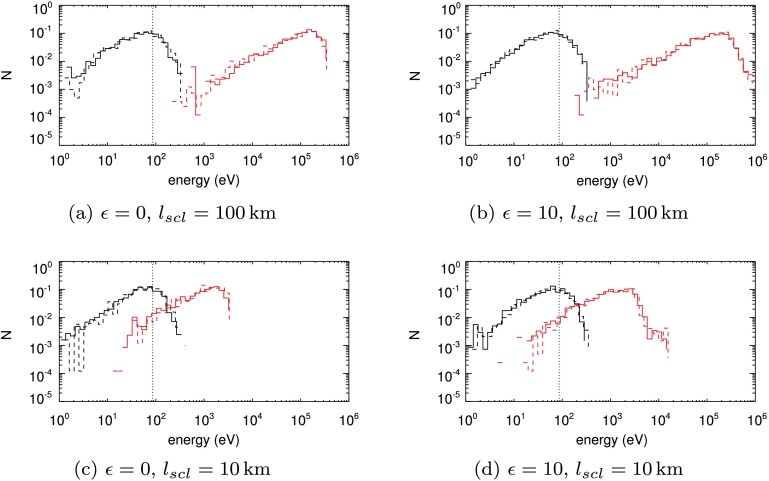
Energy spectra of our test-particle calculations of the Case 2 initial (black) and final energies (red) for both electrons (solid line) and protons (dashed line). The left column represents orbits beginning when $\epsilon=0$, while the right column shows $\epsilon =10$. The spectra in the top row are calculated at normalising length scale ${l_{\mathrm{scl}}}=100~\mbox{km}$, while those in the bottom row are for ${l_{\mathrm{scl}}} =10~\mbox{km}$.

Another way to evaluate the properties of the kinetic energies of the test particles is to form energy spectra, where the orbit energies are binned and the number of orbits in each bin is presented. To show how this energy spectrum changes over time, we use the initial conditions of Case 2. Figure 6 contains example spectra at the start and at the end of the orbit lifetimes for four different cases. The top row (Figures 6a and 6b) illustrates the initial and final energies of electrons and protons at two different stages of the global eruption model, $\epsilon=0$ and $\epsilon=10$, respectively. The bottom row (Figures 6c and 6d) shows the initial and final energy spectra at the same stages, but with a tenfold reduction in the global model length scale (*i.e.* ${l_{\mathrm{scl}}}=10~\mbox{km}$).

Electron and proton final spectra are a close match in all cases in Figure 6. At the original length scale used in earlier experiments (${l_{\mathrm{scl}}}=100~\mbox{km}$), all stages of the global eruption model recover large energy gains. The final energy spectrum terminates abruptly at the high energy end when $\epsilon=0$ (Figure 6a), where there are fewer particles above $0.4~\mbox{MeV}$ than when $\epsilon=10$. Figure 6b again shows that the later stages of the global eruption model allow particles to achieve MeV energy gains or higher, with a bump on the high-energy tail of the final distribution.

The effect of reducing the length scale of the experiment by a factor of 10 is clear from Figures 6c and 6d, which show that before termination, the particle orbit spectra now gain less kinetic energy over their lifetimes. The final energy spectra peak at approximately 1 – 2 keV, almost exactly a hundredfold reduction in the peak value for the spectra in Figures 6a and 6b. At the beginning of the eruption ($\epsilon=0$) at the smaller length scale (${l_{\mathrm{scl}}}=10~\mbox{km}$), no particles achieve kinetic energies greater than $4~\mbox{keV}$ in Figure 6c. Later stages of this eruption increase the peak kinetic energy value to approximately $20~\mbox{keV}$, as shown in Figure 6d. This increase in peak kinetic energy between early and latter stages of the experiment strongly resembles the increase seen in the case where the experiment used a higher value of ${l_{\mathrm{scl}}}$.

## Analysis

In this experiment, our aim is to study particle acceleration in a simple analytical reconnection model in the absence of topological features commonly associated with reconnection. While our findings can be compared to those recovered by other test-particle studies of various models of magnetic reconnection (discussed in Section 4.1), several aspects of our results also bear a strong resemblance to certain morphological features observed during typical (two-ribbon) solar flares. We also include a brief discussion of these aspects in Section 4.2.

### Comparison with Topological Reconnection Models

Before placing our findings in context with other models of magnetic reconnection, we would first like to emphasise that the nature of magnetic reconnection significantly changes when one moves from a two- to a three-dimensional picture. The reduced degree of freedom in 2D restricts magnetic reconnection to only take place at an X-type null point, where field lines are cut and pasted together in pairs and the electric field is perpendicular to the magnetic field, thus no acceleration due to parallel electric fields is possible. In 3D, magnetic reconnection occurs within a finite volume where the component of the electric field parallel to the magnetic field is non-zero and through which magnetic connectivity changes continuously (see *e.g.* Schindler, Hesse, and Birn, 1988; Hesse and Schindler, 1988; Biskamp, 2000; Priest and Forbes, 2000; Birn and Priest, 2007, and references therein). Thus, in contrast to 2D scenarios, a key particle acceleration mechanism is the parallel electric field in 3D. We therefore limit the scope of our comparison here to solely consider fully 3D magnetic reconnection models.

Previous models of particle acceleration at 3D magnetic reconnection sites are underpinned by the specific topology of the reconnection site itself. 3D magnetic null points are likely sites of reconnection that are known to be abundant in the solar atmosphere (*e.g.* Longcope and Parnell, 2009). The magnetic field configuration in the local vicinity of each null characteristically comprises specific structures, known as the fan or separatrix surface (a surface of field lines radiating out from or in towards the null) and spine (where field lines asymptotically approach or extend away from the null along a single axis). When considered in isolation (depending on how the reconnection itself proceeds), various effects including drifting, mirroring, and acceleration have been studied in reference to both protons and electrons in a number of different experiments (*e.g.* Dalla and Browning, 2005, 2006, 2008; Stanier, Browning, and Dalla, 2012). In our experiment, we see no evidence of mirroring and a minimal impact of drifts. Because there are no significant or dramatic changes in magnetic field strength, we find that rapid direct acceleration by the local electric field dominates the particle motion. Some models of acceleration at 3D nulls have shown a tendency to efficiently accelerate protons over electrons (Guo *et al.*, 2010). In our approach both electrons and protons are equally efficiently accelerated. The structures associated with 3D magnetic nulls must, in reality, be embedded in and form part of the local magnetic environment in the solar corona (in a number of possible configurations). Observational examples of nearly circular post-flare loops (*e.g.* Masson *et al.*, 2009) use potential field extrapolations to map the loop locations to a local separatrix dome formed from the fan plane of an overlying coronal null. Baumann, Haugbølle, and Nordlund (2013) modelled a similar configuration using a novel combination of PIC and magnetohydrodynamic (MHD) approaches and revealed that the underlying particle acceleration was driven by direct acceleration from interaction with the local reconnection electric field. We would also note the discussion of Pontin, Galsgaard, and Démoulin (2016) regarding the need for a self-consistent model of particle acceleration at a null-point current sheet. As with 3D null-point reconnection models, we also use a simplified analytical model as a crude first step towards predicting energetic particle locations or impact sites in a flux rope eruption event.

In addition, reconnection and particle acceleration have also been studied in detail at magnetic separators. These are special magnetic field lines that link pairs of magnetic nulls (marking the intersection of the fan planes of both nulls). Like null points, separators are also preferred sites for current sheet formation and reconnection, and have been inferred as sites that undergo magnetic reconnection in the solar corona (*e.g.* Longcope *et al.*, 2005). A series of recent articles (Threlfall *et al.*, 2015, 2016a) has investigated the implications of separator reconnection upon local particle dynamics, using both simple analytical and complex numerical models of several idealised isolated separator reconnection configurations. As here, direct acceleration by local parallel electric field dominated the particle motion. Impact sites were seen to closely align with the spines and local separatrix surfaces of each null threaded by the separator. Drifts and magnetic mirroring were also identified, but played a lesser role than in the isolated 3D null cases. Electrons were typically accelerated towards two specific impact sites associated with the spines of one null in the system. Accelerated proton impact sites were aligned with the spines of the other null, while the protons themselves took fractionally longer to accelerate. Our present investigation also recovers impact sites at opposite corners of the domain depending on particle charge. A model of separator reconnection that is embedded in a coronal environment is needed to provide a direct comparison with our model (and indeed the embedded 3D null models described earlier). As an aside, we also note that the scaling of energy gains described in detail in Threlfall *et al.* (2016a) is also present in our results. Any scale-free model of magnetic reconnection where particle energy gains are due to a (field-aligned) potential difference may be rescaled without recalculation of the particle orbits, according to

$$\Delta= \int E_{\|}\, ds=\frac{{l_{\mathrm{scl}}}^{2}{b_{\mathrm{scl}}}}{{t_{\mathrm{scl}}}} \int {\tilde {E_{\|}}\,d\tilde{s}}=\frac{{l_{\mathrm{scl}}}^{2}{b_{\mathrm{scl}}}}{{t_{\mathrm{scl}}}}\tilde { \Delta}, $$

where $\Delta$ is the change in energy, and barred quantities represent the normalised values of their counterparts. In Section 3.3 we describe how a tenfold reduction in length scale yields a hundredfold reduction in energy gained by the particle orbits. This is exactly as expected from this simple expression derived in Threlfall *et al.* (2016a). Thus scale-free models like this one allow for the coverage of larger ranges of parameter space without additional computational effort.

The final type of models that we broadly compare to here are reconnecting coronal loop models. Turkmani *et al.* (2005, 2006) inserted test particles into a magnetic cylinder that modelled a coronal loop subjected to photospheric driving, which built up fragmented current sheets throughout the volume. The peak absolute electric field within the current sheets reached approximately $900~\mbox{V}\,\mbox{m}^{-1}$, and consequently, both protons and electrons were able to achieve up to $100~\mbox{GeV}$ energies. Energised particles in the simulations were either trapped by repeated mirroring due to interaction with multiple electric field regions or exited the loops via the loop footpoints. Our model is able to better control the peak electric field values by the use of a single monolithic electric field at a single broad location, which causes all orbits to be accelerated to some degree. With only a single electric field region, this electric field mirroring (also seen in *e.g.* Threlfall *et al.*, 2016b, for simulations of a full active region) cannot be studied. A future extension of our work should aim to reduce the size of the reconnection region (for example using the parameters $L_{x}$ and $L_{z}$, which were chosen here to match the original model of Hesse, Forbes, and Birn, 2005) and include complexity through the insertion of additional reconnection regions.

A similar, more recent model of reconnecting coronal loops (Gordovskyy *et al.*, 2014) also shows high-energy proton and electron precipitation towards the loop footpoints. These authors recover a high-energy power-law tail using initially Maxwellian particle energies, likely due to the reconnection of multiple fragmented current sheets, again implying that our model lacks the appropriate size and sporadic nature of the electric field using the parameter values specified here.

While our simple model certainly has drawbacks, it also has advantages over these models. For example, it is unclear whether the reconnection in coronal loop models takes place at or in the presence of specific topological features (*e.g.* nulls or separators) or geometric features (*e.g.* quasi-separatrix layers, or QSLs, see Titov, Hornig, and Démoulin, 2002). In our case, we can definitively state that the reconnection modelled here is not associated with separators, separatrix surfaces, or magnetic nulls in any way.

### Qualitative Comparison with Flare Observations

Several facets of the results presented earlier are reminiscent of motions, associated or underpinned by reconnection, that are observed during flares. A comprehensive overview of flare observations can be found in *e.g.* Fletcher *et al.* (2011). By comparing our results with flare observations, we do not seek to suggest that this model underpins or models a solar flare, but merely that it highlights some interesting similarities between this simple model and aspects of flare observations. In this discussion, we refer to the classic two-ribbon flare picture (discussed in *e.g.* Savcheva *et al.*, 2016, and based on references therein). Two types of apparent motions are prevalent in observations of such flares. One type is a fast elongation (often termed a “zipper” motion) running parallel to the PIL during the impulsive phase of these flares. The other type is a gradual expansion of the ribbons perpendicular to the PIL during the decline phase of these flares.

Our particle results suggest that both types of motions may be present in our model. The footpoints of our initial arcade clearly separate over time, as seen in Figure 1. The particle impact sites themselves (seen in Figure 3) track this separation, with the highest energy impact sites also steadily diverging from the lowest, as the footpoints move apart. This divergence from one to two impact sites would likely generate observational signatures suggesting an apparent motion perpendicular to the PIL. Using the original normalising length and timescale in our experiment (${l_{\mathrm{scl}}}=100~\mbox{km}$, ${t_{\mathrm{scl}}}=100~\mbox{s}$), we estimate an apparent velocity of $0.15~\mbox{km}\,\mbox{s}^{-1}$ for this motion, well below typical speeds recovered by observations. Flare footpoint widening observations are not always definitive, however, with footpoint widening (and indeed contraction) varying from flare to flare across a range of speeds (*e.g.* Fletcher and Hudson, 2002). Varying the parameters in our model (such as using an appropriate non-linear as opposed to linear function of $\epsilon(t)$) would lead to more rapid motions, as the approximate locations of highly accelerated particle impact sites at given times would be affected.

We also recover evidence of an apparent motion that runs parallel to the PIL. Over time, the asymmetry (in $x$) of final particle positions of highly accelerated orbits continues to develop and can clearly be seen in Figure 3. Observations of $H\alpha$ and hard X-ray footpoint sources sometimes move along extreme ultraviolet (EUV) flare ribbons rather than away from the PIL. These locations (and their apparent motions) are known to represent intense energy deposition in the lower solar atmosphere (*e.g.* Hudson, Wolfson, and Metcalf, 2006). By averaging the final $x$-position of orbits that achieved the largest energy gains, we again broadly estimate an apparent velocity of the impact sites of this population of approximately $0.2~\mbox{km}\,\mbox{s}^{-1}$ (when ${l_{\mathrm{scl}}}=100~\mbox{km}$, ${t_{\mathrm{scl}}} =100~\mbox{s}$). This speed is also much slower than the typical velocities of apparent PIL-parallel motions recovered by observations, but as before, it is dependent on the parameter values and box size we have chosen.

Our apparent parallel and perpendicular velocities appear much too low when compared to those uncovered during flare observations. We would once again note, however, that we have chosen model parameters and to non-dimensionalise our system without any consideration of modelling a solar flare. Our model can be rescaled in a number of ways, for example through ${l_{\mathrm{scl}}}$, ${b_{\mathrm{scl}}}$, and ${t_{\mathrm{scl}}}$ (or indeed through the parameters $L_{x}$, $L_{y}$, and $L_{z}$) in order to yield speeds of apparent motions that are much closer to those found from solar flare observations. The factor 0.2 is also arbitrary, as is the functional dependence of $\epsilon$ on $t/\tau$.

In addition to velocities, there are other minor similarities between our model results and solar flare observations. For example, we note that our model arcade is initially sheared. The probability of an active region or arcade structure to produce a flare has often been linked to the buildup of shear along the polarity inversion line (*e.g.* Hagyard *et al.*, 1984; Leka and Barnes, 2007; Qiu, Gary, and Fleishman, 2009). It is also interesting to note that here the footpoints of the magnetic field do not actually move (since there is no plasma flow on the base), despite the apparent motions of particle impact sites. Equation (6) confirms that the photospheric magnetic field in this model is fixed, while there are significant changes to the coronal magnetic field. This confirms that apparent motions of the particle impact sites may be important tools for unraveling the behaviour of coronal magnetic fields (and in turn inferring the nature of magnetic topology and reconnection) during solar flares (*e.g.* Masson *et al.*, 2009).

## Conclusions and Future Work

In this article, we have adapted a scale-free model of a magnetic flux-rope eruption, originally proposed by Hesse, Forbes, and Birn (2005), and employed it to study particle acceleration in a 3D magnetic reconnection configuration that crucially does not depend on specific topological features, such as magnetic nulls and separators. In this model, the eruption of the magnetic flux rope and the formation of helical magnetic field lines allow electron and proton orbits to repeatedly re-enter the reconnection region, whereupon they receive strong direct acceleration from the electric field that is associated with the magnetic reconnection. The impact sites of these particles move over time, according to which particle species is being considered; both electrons and protons are ultimately able to achieve the same energies in this model. The model itself is inherently scale free, allowing us to suggest how the final orbit energy gains recovered would change not only over the course of the flux-rope eruption model, but also scale with different normalisation (with a peak energy gain of 2 MeV or 20 keV depending on the parameter regimes used here) according to the formula derived in Section 4.1. Our orbit calculation results also bear a notable resemblance to aspects of solar flare observations, although we make no attempt to model such a scenario.

A natural first extension of this work would be to investigate the presence of QSLs, which are geometric (rather than topological) features where reconnection and particle acceleration are thought to take place. It is also worth exploring whether we can reduce the reconnection region size and add more reconnection sites (in the manner seen in simulations of reconnection at fragmented current sheets within coronal loops), and how this would affect the flux rope formation in this model. In line with previous work, a more extended next step would be to attempt to model this eruption using high-resolution MHD simulations. The localised magnetic reconnection that takes place in this model results from a specific magnetic perturbation, introduced to allow the flux rope to erupt. It would be of interest to see whether or how (for example) footpoint motions or an initially non-equilibrium field configuration might build up current layers within the original arcade structure. How these current layers would then dissipate (through reconnection), and whether the configuration would continue to be devoid of magnetic null points or separators, are also open questions.
